# Silver nanoparticles defeat p53-positive and p53-negative osteosarcoma cells by triggering mitochondrial stress and apoptosis

**DOI:** 10.1038/srep27902

**Published:** 2016-06-13

**Authors:** Dávid Kovács, Nóra Igaz, Csilla Keskeny, Péter Bélteky, Tímea Tóth, Renáta Gáspár, Dániel Madarász, Zsolt Rázga, Zoltán Kónya, Imre M. Boros, Mónika Kiricsi

**Affiliations:** 1 Department of Biochemistry and Molecular Biology, University of Szeged, Középfasor 52, H-6726, Szeged, Hungary; 2 Department of Applied and Environmental Chemistry, University of Szeged, Rerrich Béla tér 1. H-6720, Szeged, Hungary; 3 Department of Biochemistry, Faculty of Medicine, University of Szeged, Dóm tér 9. Szeged, H-6720, Hungary; 4 Department of Pathology, University of Szeged, Állomás utca. 2. H-6720, Szeged, Hungary; 5 MTA-SZTE Reaction Kinetics and Surface Chemistry Research Group, Rerrich Béla tér 1, H-6720, Szeged, Hungary; 6 Institute of Biochemistry, Biological Research Center of the Hungarian Academy of Sciences, Temesvári krt. 62, H-6726, Szeged, Hungary

## Abstract

Loss of function of the tumour suppressor p53 observed frequently in human cancers challenges the drug-induced apoptotic elimination of cancer cells from the body. This phenomenon is a major concern and provides much of the impetus for current attempts to develop a new generation of anticancer drugs capable of provoking apoptosis in a p53-independent manner. Since silver nanoparticles (AgNPs) possess unique cytotoxic features, we examined, whether their activity could be exploited to kill tumour suppressor-deficient cancer cells. Therefore, we investigated the effects of AgNPs on osteosarcoma cells of different p53 genetic backgrounds. As particle diameters might influence the molecular mechanisms leading to AgNP-induced cell death we applied 5 nm and 35 nm sized citrate-coated AgNPs. We found that both sized AgNPs targeted mitochondria and induced apoptosis in wild-type p53-containing U2Os and p53-deficient Saos-2 cells. According to our findings AgNPs are able to kill osteosarcoma cells independently from their actual p53 status and induce p53-independent cancer cell apoptosis. This feature renders AgNPs attractive candidates for novel chemotherapeutic approaches.

Despite significant advancements in understanding tumour development and progression at the cellular and molecular levels, managing metastatic and recurrent cancers still remains an overwhelming task^1^. Over the past decade promising alternative strategies for treating several forms of cancers have been developed, many of these are based on the unique physicochemical and biological properties of organic and inorganic nanoparticle systems^2^,^3^,^4^.

Silver nanoparticles (AgNPs) have been found to possess strong antimicrobial properties^5^, yet their intrinsic cytotoxic and antitumour activities have been demonstrated reliably only a few years ago^6^,^7^,^8^,^9^. Recent studies on rats with Pliss lymphosarcoma, on Dalton’s ascites tumour model and on breast cancer xenograft bearing mice confirmed that AgNPs inhibit the growth of tumour tissues *in vivo*^10^,^11^,^12^. AgNPs caused cancer cell apoptosis, cell cycle arrest and decreased viability of colon cancer cells *in vitro*^13^,^14^,^15^,^16^. A very elegant study on a multidrug resistant cancer model revealed an enhanced antitumour effect of AgNPs functionalized with cell penetrating peptides^17^, suggesting that surface modification of the nanoparticles may increase their efficiency and cancer cell specificity *in vivo*.

Owing to these cytotoxic properties and due to the broad application of silver nanoparticles, the molecular background of AgNP-induced cell death has been intensively examined in various models^18^,^19^,^20^,^21^. Comprehensive toxicological studies indicated that AgNPs kill cells through a Trojan-horse type mechanism, suggesting that the intracellularly accumulated nanoparticles release toxic silver ions^22^. Those ions induce the generation of reactive oxygen species (ROS) thereby the redox homeostasis of the cells becomes unbalanced^23^,^24^. The reactive species then target biomolecules and cellular organelles inducing membrane disorder, cytoskeletal disruption and genotoxic lesions, which altogether contribute to initiation of the apoptotic process^25^,^26^,^27^. It is still debated whether the particles *per se*, the amount of silver ions or the combination of these drive AgNP toxicity. It is generally accepted that AgNP-induced cell death is primarily the result of oxidative damage, however, various reports indicated that the observed oxidative stress is independent of the toxicity of silver ions^28^,^29^.

A few recent publications suggested that the relative surface of the nanoparticles is related to their silver ion releasing capability resulting in higher cytotoxic propensity of the smaller nanoparticles^30^,^31^,^32^. It has been reported that 5 nm AgNPs were more toxic compared to 20 nm and 50 nm particles in four different cell lines^33^. Smaller AgNPs induced interleukin 8 release and ROS generation with a higher degree than larger nanoparticles^24^. Whereas in another study^34^ elevated release of lactate dehydrogenase and lower cell viability were observed due to 100 nm sized particles compared to treatments with 10 nm and 50 nm sized AgNPs. Considering the sometimes confusing information available on the contribution of nanoparticle size to the cytotoxic features it is conceivable that smaller and larger particles target and activate diverse cellular mechanisms leading to cell death.

The studies mentioned above provided evidence on the antitumour effect of AgNPs, nonetheless, cancer cells are equipped with an ample set of mechanisms to overcome anticancer agent-induced cellular stress. One of the most favoured strategies to escape cell death is the inactivation of tumour suppressors like p53. Normally, p53 serves as a multifunctional platform to overcome cellular impairments such as oxidative stress, hypoxia, heat shock and activation of oncogenes, however, it is accepted that DNA damage is the major activator of p53-dependent signalling pathway^35^. Activation of p53 is associated with its accumulation resulting in enhanced transcription of p53 responsive genes, which mediate cell cycle arrest and most importantly, initiate apoptosis^36^,^37^,^38^. As p53 is mainly activated by nuclear impairments, the most frequently applied DNA targeting chemotherapeutic drugs such as cisplatin, doxorubicin and etoposide, trigger p53-mediated cell death^39^. Nearly 50% of all human cancers have been characterised by impaired p53 function which attenuates therapeutic efficacy^40^. Since p53−/− cancers often manifest chemoresistance towards anticancer drugs, a strong correlation between p53 status and therapeutic outcome can be established^40^,^41^. Cancer cells are able to get rid of the tumour suppressing effect of p53 by various mechanisms. For instance, point mutations in the DNA binding domain may lead to protein variants which are not able to bind to the regulatory regions of target genes^42^,^43^. Another possible way to eliminate p53 is the deletion of its chromosomal locus from the genome. Although inactivation of p53 renders the drug-induced elimination of cancer cells rather difficult, p53-independent apoptotic pathways could be exploited in drug design and development. For example, targeting mitochondria might be promising alternative, as the damage of its structure leads directly to apoptotic cell death through disruption of mitochondrial membrane potential, cytochrome c release and apoptosome assembly. Furthermore, ER stress or autophagy can also induce cancer cell apoptosis in a p53-independent manner^44^,^45^,^46^,^47^.

As lesions in the p53 tumour suppressor gene are the most commonly detected genetic alterations in human tumours^48^ our main goal was to investigate whether the actual p53 status of cancer cells would influence the AgNP-induced molecular mechanisms. We hypothesized that AgNP-provoked cell death could be realised both by p53 activation and also by the stimulation of p53-independent events. To prove this hypothesis, we used a model system containing two osteosarcoma cancer cell lines – wild type p53-expressing U2Os and p53 null mutant Saos-2 cells – and we examined the cellular events following AgNP administration. To address the issue of particle size dependency of AgNP-induced cell death we applied biocompatible citrate-coated 5 nm and 35 nm sized AgNPs on both cell lines. In this study we show, for the first time, that although AgNP treatments activated p53 in p53 containing osteosarcoma cells, the primary action of AgNPs is the induction of mitochondrial stress which drives cancer cell apoptosis in a p53-independent manner.

## Results

### Synthesis and characterization of citrate-coated AgNPs

**Figure 1:**
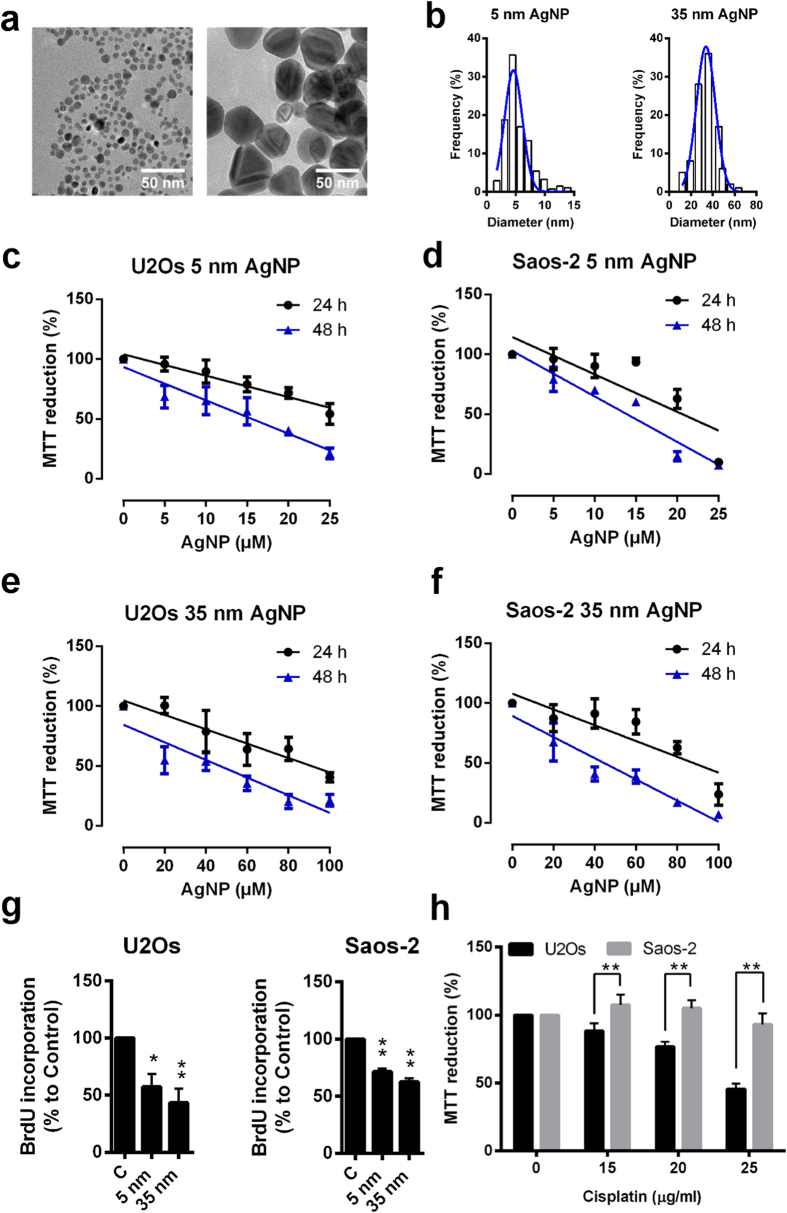
AgNPs decrease the viability of p53-expressing U2Os and p53-deficient Saos-2 cells with the same extent. (**a**) Representative TEM micrographs of differently sized citrate-coated AgNPs. (**b**) Size distribution of nanoparticles was determined by TEM image analysis. (**c–f**) Viability of p53-expressing U2Os and p53-deficient Saos-2 cells was determined by MTT assay after 24 h and 48 h of 5 nm or of 35 nm sized AgNPs administrations. Each point represents the mean value with error (SD) of three independent experiments. (**g**) Decreased proliferating activity of U2Os and Saos-2 cells was observed after 24 h AgNP treatments as determined by measuring BrdU incorporation. *P ≤ 0.01, **P ≤ 0.001, Dunnett’s multiple comparisons test. (**h**), Cisplatin treatment decreased the viability of wild type p53-expressing U2Os but did not influence p53-deficient Saos-2 viability. **P ≤ 0.001, Sidak’s multiple comparisons test.

In order to analyse the anticancer activity of AgNPs, we synthetized quasi-spherical citrate-coated silver nanoparticles of different sizes. Biocompatible sodium citrate was applied as a reducing and coating material. The method we used proved to be suitable to synthesise stable solutions of AgNPs in desired sizes (Fig. 1a). UV-VIS spectra of the obtained colloidal AgNP solutions have a characteristic peak maximum around 400 nm, which can be attributed to surface plasmon resonance (Fig. S1). Size distribution of the as-prepared particles was assessed by transmission electron microscope (TEM) image analysis and was verified by Dynamic Light Scattering (DLS) measurements. TEM and DLS confirmed the successful production of quasi-spherical AgNPs of 5 nm and 35 nm average diameters (Fig. 1b, Fig. S2a,b, Table S1). Nanoparticle samples containing smaller AgNPs have a narrow size distribution (approximately 2–15 nm), however, larger AgNPs represent a broad range of particle diameter (approximately 10-70 nm). Stability of the AgNPs was tested by DLS measurements after 24 h ageing in serum free DMEM or in DMEM supplemented with 10% FBS. In accord with other studies on nanoparticle stability^49^, AgNPs formed aggregates in serum free DMEM (Fig. S2c,d), however nanoparticles diluted in the same medium supplemented with 10% FBS proved to be stable (Fig. S2e,f). Therefore, upon AgNP treatments nanoparticles were dissolved in full culture medium containing FBS. Zeta potential measurements indicated that citrate-coated nanoparticles have negatively charged surface both in aqueous solution and in different culture media (Table S1).

### AgNPs kill osteosarcomic cancer cells with different p53 statuses

Wild type p53-expressing U2Os and p53-deficient Saos-2 osteosarcoma cells were treated with 5 and 35 nm sized AgNPs in various concentrations (5–100 μM) for 24 or 48 h and cell viability was determined by MTT assay (Fig. 1c–f). Smaller nanoparticles possessed higher cytotoxic properties than bigger ones after 24 h exposition: 5 nm AgNPs decreased cell viability already at 15–20 μM concentration while significant loss of metabolic activity was achieved only at 40 μM concentration upon 35 nm AgNP treatment. 48 h administration of nanoparticles decreased cell viability with a higher degree compared to 24 h treatments, demonstrating the time dependent cytotoxicity of AgNPs. IC_50_ values corresponding to the two AgNP sizes for both the 24 h and 48 h treatments were calculated using the data obtained from the MTT assays (Table 1). As only minor differences were detected in the IC_50_ values of U2Os and p53-deficient Saos-2 cells our results suggest that AgNPs can also induce cancer cell death in a p53-independent manner.

In order to investigate whether AgNPs with smaller and bigger sizes kill cancer cells by attacking different or identical cellular targets we treated osteosarcomic cells with AgNPs in concentrations approximating the calculated IC_50_ values (i.e. 5 nm AgNPs were applied in 20 μM, whereas 35 nm AgNPs in 85 μM concentrations). Decreased proliferating activities of U2Os and Saos-2 cells were detected upon both sized AgNP administrations. The S-phase dependent incorporation of BrdU demonstrated the antiproliferative effect of the nanoparticles in the selected concentrations (Fig. 1g).

To compare the anticancer activity of AgNPs with a clinically applied chemotherapeutic agent on p53-expressing and p53-deficient osteosarcoma cells, U2Os and Saos-2 cells were treated with cisplatin in various concentrations and cell viabilities were determined after 24 h. Cisplatin in the applied concentrations did not influence the viability of p53-deficient Saos-2 cells, while wild type p53-expressing U2Os cells showed higher sensitivity to cisplatin treatments. (Fig. 1h).

**Figure 2:**
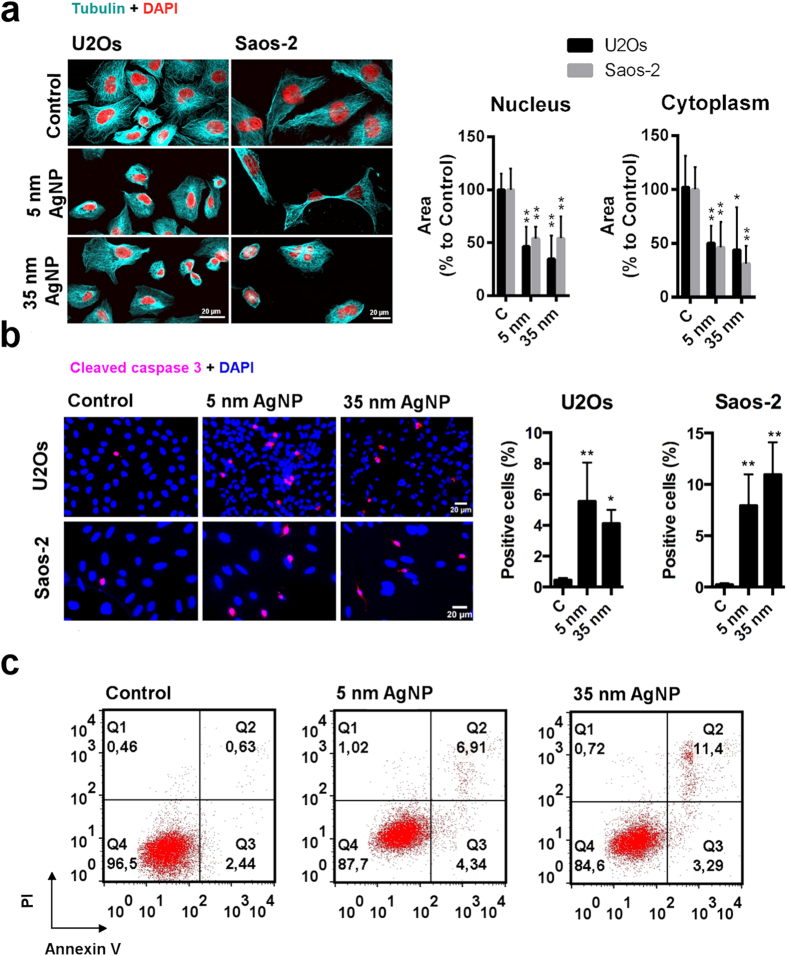
AgNPs induce apoptosis in p53-expressing U2Os and p53-deficient Saos-2 cells. (**a**) Confocal microscopic visualisation of tubulin fibres and nuclei show apoptotic morphology of U2Os and Saos-2 cells after AgNP treatments. The area of the nuclei and of cytoplasms was significantly reduced in AgNP-treated U2Os and Saos-2 cells compared to those of the untreated cells, respectively. *P ≤ 0.05, **P ≤ 0.01, Tukey’s multiple comparisons test. (**b**) 5 nm and 35 nm sized AgNP treatments increased the number of cells containing cleaved caspase 3. The number of cells containing cleaved caspase 3 was quantified after AgNP treatments and expressed as % of total cell number. *P ≤ 0.05, **P ≤ 0.01, Tukey’s multiple comparisons test. (**c**) Increased ratio of AnnexinV- and PI-positive cells was observed after 24 h AgNP treatments in p53-deficient Saos-2 cells. Representative dot plots of flow cytometry data show that both 5 nm and 35 nm AgNP treatments induced apoptosis.

Both wild type p53-expressing U2Os and p53-deficient Saos-2 osteosarcoma cells showed decreased cell viability and loss of metabolic activity upon treatments with either 5 or 35 nm sized AgNPs. The observed cancer cell death provoked by AgNPs could be the result of the apoptotic execution. In order to confirm the involvement of apoptosis in AgNP-provoked cellular events, we performed first a morphological analysis on 5 nm and 35 nm sized AgNP-treated cells. This examination revealed disrupted tubulin fibres, shrunken nuclei and reduced cytoplasmic volume in both osteosarcoma cell lines (Fig. 2a).

Induction of apoptosis was verified by immunostaining U2Os and Saos-2 cells with cleaved caspase 3 specific antibody after treatments with 20 μM of 5 nm and with 85 μM of 35 nm sized AgNPs for 24 h (Fig. 2b). Cleaved caspase 3 positive cells were counted and expressed as the percentage of total cell number. 5 nm and 35 nm sized AgNPs induced caspase 3 activation with the same extent in both osteosarcoma cell lines.

Finally, we wanted to prove that the p53-independent caspase 3 activation is exclusively the result of apoptosis and necrotic cell death does not contribute to the previously observed cytotoxicity. Therefore, we treated Saos-2 cells with 20 μM of 5 nm and with 85 μM of 35 nm sized AgNPs for 24 h, and the percentage of apoptotic (Q2 + Q3) and necrotic (Q1) cells was quantified by flow cytometry using AnnexinV/PI assay. Representative dot plots of flow cytometry data (Fig. 2c) show that both 5 nm and 35 nm AgNP treatments induced apoptosis in Saos-2 cells.

### AgNPs are taken up by osteosarcoma cells

**Figure 3:**
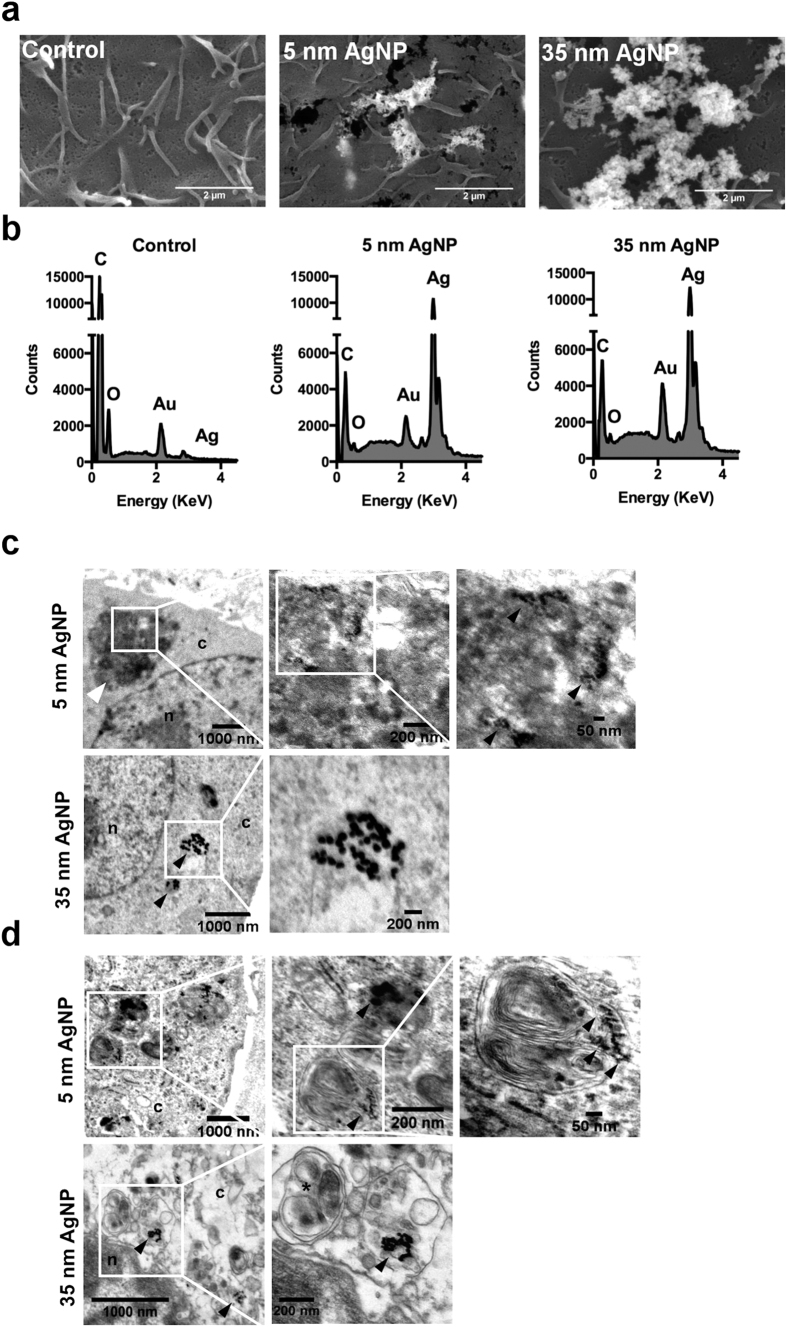
AgNPs attach to the cell surface and are internalised by osteosarcoma cells. (**a**) SEM micrographs show 5 nm and 35 nm sized AgNPs attached to the membrane of U2Os cells. (**b**) EDS spectra of AgNP-treated U2Os cells. Au peaks arise due to gold coating used in SEM sample preparation. TEM micrographs of AgNP-treated U2Os (**c**) and Saos-2 cells (**d**). White arrow indicates dense AgNP containing endosome. Black arrows point to intracellular AgNPs. c – cytoplasm, n – nuclei. Asterisk indicates membrane coated cellular organelles.

Aggregated AgNPs were observed on the surface of treated U2Os cells by SEM indicating the attachment of particles to the cell membrane (Fig. 3a). The chemical composition of the samples determined by electron microscopy using EDS detector further verified the presence of silver nanoparticles (Fig. 3b). Both sized AgNPs appeared not only on the cell surface but were also internalized within U2Os and Saos-2 cells. According to TEM micrographs, AgNPs localized in membrane coated multivesicular and multilamellar bodies and were also dispersed in the cytoplasm. No nanoparticles were detected in the nuclei nor within mitochondria (Fig. 3c–d). In U2Os cells 35 nm sized AgNPs were localized both in membrane coated bodies and dispersed in the cytoplasm, while 5 nm sized spherical objects were identified as AgNPs in electron dense endosomes (Fig. 3c). In Saos-2 cells both sized AgNPs were deposited in vesicular bodies. Interestingly, in these cells, membrane coated cellular organelles were also observed, potentially indicating the involvement of autophagy related mechanisms in AgNP-induced toxicity (Fig. 3d).

### AgNPs stimulate p53 signalling

To test whether the p53-dependent signalling pathway is activated upon AgNP administration, we studied both the protein level and the transactivating capability of p53. Additionally, the relative expression of p53 target genes were also investigated.

**Figure 4:**
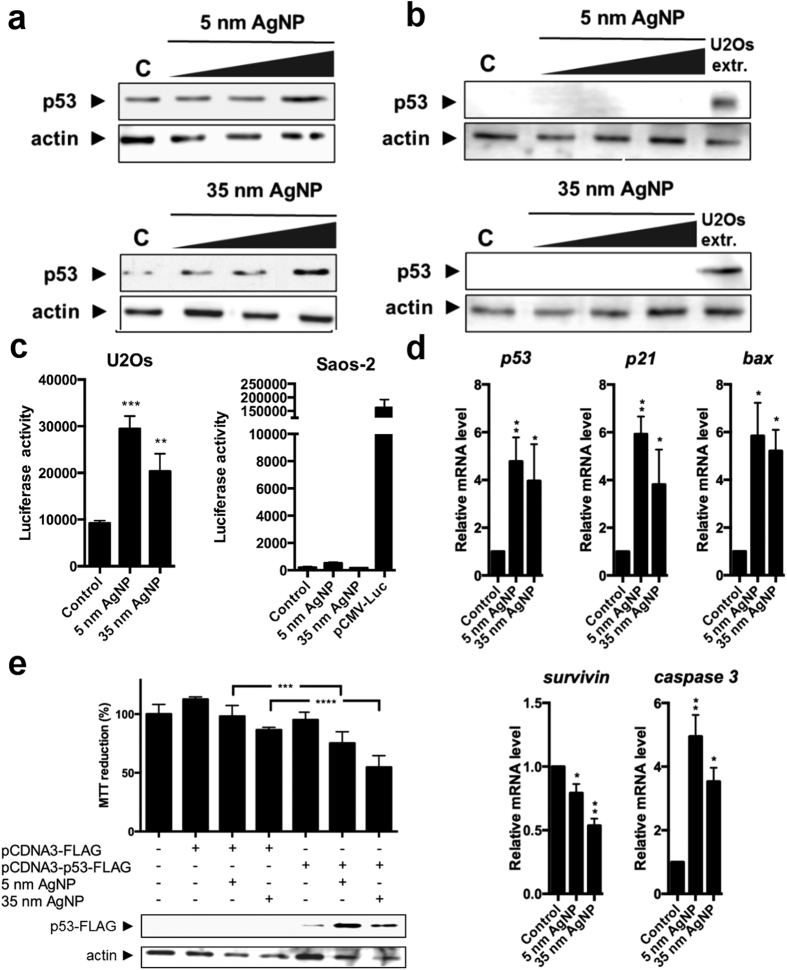
AgNP treatments activate the p53-dependent signalling pathway. (**a**) Representative western blots of 0; 10; 15; 20 μM of 5 nm and 0; 40; 60; 85 μM of 35 nm sized AgNPs treated U2Os cells. Both sized AgNP treatments stabilized p53 protein. (**b**) Saos-2 cells do not contain any p53 protein. U2Os extract was used as a p53 protein containing positive control. (**c**) AgNP treatments induce the p53 responsive mdm2 promoter activity in U2Os cells. No activity was detected in Saos-2 cells. pCMV-Luc was used as a positive control of Saos-2 transfections. **P ≤ 0.01, ***P ≤ 0.001 Dunnett’s multiple comparisons test. (**d**). Elevated levels of p53, p21, bax, caspase 3 and decreased amount of survivin mRNAs were detected in 5 nm and 35 nm AgNP-treated U2Os cells. *P ≤ 0.05, **P ≤ 0.01, Sidak’s multiple comparisons test. **(e)** AgNP treatments kill ectopically p53-expressing Saos-2 cells with a higher degree than empty vector pHC624 containing control cells. Using western blot successful ectopic expression was verified. ***P ≤ 0.001, ****P ≤ 0.0001, Tukey’s multiple comparisons test.

For this, we treated U2Os cells with 0; 10; 15; 20 μM of 5 nm and 0; 40; 60; 85 μM of 35 nm sized AgNPs and performed western blots on whole cell lysates. The level of p53 protein increased markedly upon 20 μM of 5 nm and 85 μM of 35 nm sized AgNP treatments, suggesting the stabilization of the tumour suppressor protein (Fig. 4a). In line with expectations, western blots proved that Saos-2 cells do not contain any p53 protein (Fig. 4b).

To verify that p53 is not only stabilised but functionally activated upon AgNP expositions, U2Os cells were transfected transiently with 1 μg of pGL2-mdm2-Luc vector expressing a luciferase reporter gene under the control of the p53-responsive promoter of mdm2. 24 h after transfections, cells were treated with 20 μM of 5 nm and 85 μM of 35 nm sized AgNPs and luciferase activity was measured the following day. The activity of the p53-responsive promoter increased significantly, indicating elevated transactivating capability of p53 upon AgNP administrations (Fig. 4c). To prove that the observed promoter activity is exclusively dependent on p53, a similar experiment was also performed on p53-deficient Saos-2 cells. As expected, no mdm2 promoter activity was detected in Saos-2 cells, while a CMV promoter-driven reporter used as positive control of the transfection showed high activity in these cells (Fig. 4c). Due to AgNP administrations, significantly elevated mRNA levels of *p53* and p53 target *p21* and *bax* genes were detected in U2Os cells by RT-qPCR. Furthermore, the transcript levels of apoptosis-related genes were also altered, as decreased *survivin* and elevated *caspase 3* mRNA levels were measured (Fig. 4d).

To examine whether the ectopic expression of p53 in the p53-deficient Saos-2 cells influences the cellular response to AgNP expositions, we transfected Saos-2 cells with FLAG-tagged p53-expressing pCDNA3 vector. Transiently transfected cells were treated with non-toxic dose of AgNPs (15 μM of 5 nm and 60 μM of 35 nm) for 24 h and subsequently viability of the cells was measured using MTT assay. Notably, while these AgNP concentrations did not influence the viability of empty vector transfected Saos-2 cells, a significant loss of viability was detected in p53-expressing cells. The expression of p53 in the transfected cells was verified by western blot on biological replicates of the experiments. Additionally, AgNP treatments stabilized the p53 protein in Saos-2 cells similarly to our previous observations on endogenous p53 in U2Os cells (Fig. 4e).

### AgNPs target mitochondria

**Figure 5:**
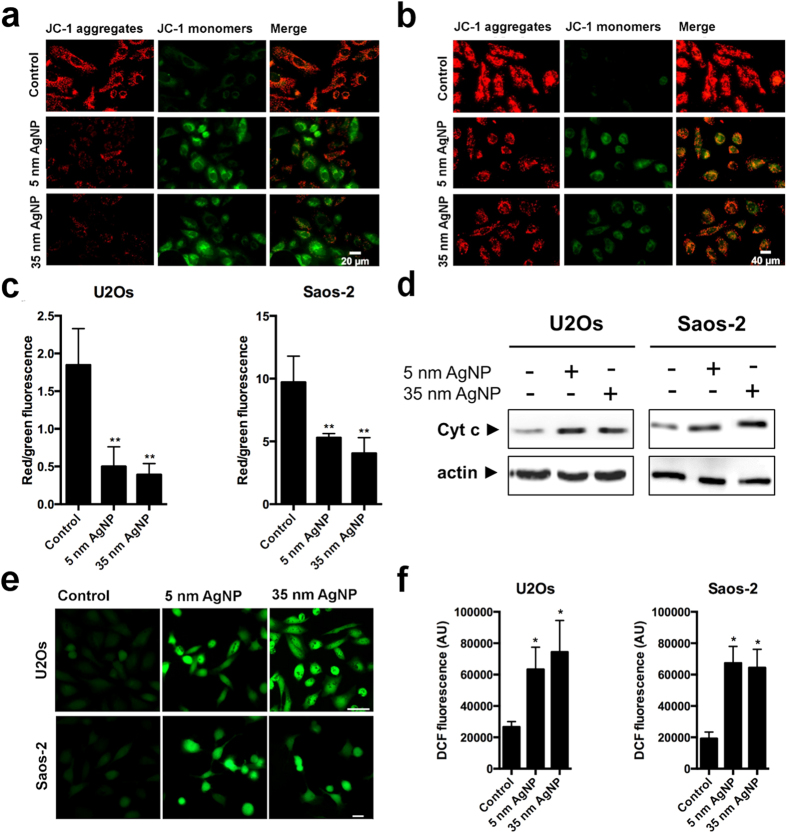
AgNP treatments induce mitochondrial stress. Decreased mitochondrial membrane potential was detected in 5 nm and 35 nm AgNPs treated U2Os (**a**) and Saos-2 (**b**) cells using JC-1 staining. (**c**) Red to green fluorescent ratio was determined by fluorescent microscopic image analysis. **P ≤ 0.01 Dunnett’s multiple comparisons test. (**d**) Elevated levels of cytoplasmic cytochrome c was detected in 5 nm and 35 nm AgNP-treated U2Os and Saos-2 cells by western blot. (**e**) Representative fluorescent microscopic images of DCFDA stained U2Os and Saos-2 cells show elevated levels of ROS upon AgNP treatments. Scale bar: 40 μm. (**f**) Fluorescent intensity of microscopic images was determined by image analysis. *P ≤ 0.0001 Dunnett’s multiple comparisons test.

The results described above demonstrated that treatments with AgNPs of both sizes activated p53 signalling. Additionally, apoptotic response was detected not only in U2Os cells but in p53 null-mutant Saos-2 cells as well, suggesting that the mediator of the AgNP-triggered cell death can also be the result of p53-independent events. To investigate whether AgNPs target mitochondria both in U2Os and in Saos-2 cells 20 μM of 5 nm and 85 μM of 35 nm sized AgNP-treated cells were stained with JC-1 and visualized by fluorescent microscopy. Microscopic images revealed that the fluorescent intensity of the red JC-1 aggregates decreased, while the intensity of the green JC-1 monomers increased upon AgNP treatments in both cell lines compared to the untreated control cells. The resulting decrease in red to green fluorescence ratio indicates the loss of mitochondrial membrane potential (Fig. 5a–c). Additionally, AgNP treatments induced cytochrome c release to the cytoplasm in both cell lines, verifying the activation of the mitochondrial apoptotic pathway (Fig. 5d). As mitochondrial dysfunction is coupled to oxidative stress, we investigated the degree of ROS generation upon AgNP treatments. In both osteosarcoma cell lines 20 μM of 5 nm and 85 μM of 35 nm sized AgNPs induced significant production of ROS further supporting mitochondrial damage (Fig. 5e,f).

## Discussion

Inactivation of tumour suppressors occurs in almost all types of human cancers^50^. Among others, the tumour suppressor p53 induces cell cycle arrest and initiates apoptosis in order to eliminate genetically unstable cells from the body, thereby preventing cancerous transformation. The lack of the cell cycle regulating and cell death initiating functions of these factors challenges the intrinsic and drug therapy-induced apoptotic elimination of cancer cells. Because of their promising features, the possible application of AgNPs in cancer therapy has recently been intensively investigated. It has already been reported that AgNPs stimulate p53-responsive gene expression^51^,^52^, suggesting that exposure of cells to AgNPs induces apoptosis via the stimulation of p53 signalling. Thus, we raised the following questions: i, are AgNPs able to kill p53-deficient cancer cells, ii, if they do, what is the mechanism of the p53-independent AgNP toxicity, iii, do these mechanisms depend on the actual size of the applied AgNPs. To answer these questions we compared the apoptotic response of p53-proficient U2Os and p53-deficient Saos-2 osteosarcoma cancer cells upon 5 nm and 35 nm sized AgNP treatments.

First, we synthesised AgNPs of smaller and larger sizes by the modified Lee-Meisel method and the characterization of the obtained nanoparticles was carried out. TEM analysis revealed a broader size distribution of the larger nanoparticle population due to the applied synthesis method.

To study the p53-dependent toxicity of AgNPs on osteosarcoma cells we treated wild type p53-expressing U2Os and p53-deficient Saos-2 cells with the synthesised 5 nm and 35 nm sized AgNPs. Similarly to the results of recent studies on the size-dependent toxicity of nanoparticles^30^,^31^,^32^ we detected that the smaller, 5 nm sized nanoparticles had stronger cytotoxic effects compared to the larger, 35 nm sized AgNPs in both cell lines. We also observed time-dependent toxicity of the AgNPs, as after 48 h of AgNP exposition the determined IC_50_ values dropped approximately to the half of those calculated for 24 h treatments (Fig. 1c–f and Table 1). Although smaller AgNPs proved to be more toxic than bigger ones on the examined cell lines, we aimed to find out whether cancer cell death induced by smaller and larger AgNPs, involves identical mechanisms or nanoparticles of different sizes achieve their toxicity through the activation of different pathways. For this purpose, we applied 5 nm and 35 nm AgNPs in concentrations corresponding to the respective IC_50_ values, in order to kill cancer cells with the same extent, and we tested the main apoptotic features in tumour cells exposed to differently sized AgNPs.

Cell viability experiments revealed that both p53-expressing U2Os and p53-deficient Saos-2 cells were killed with almost the same degree upon AgNP treatments. Although we observed minor differences in AgNP toxicity between U2Os and Saos-2 cells, AgNPs significantly reduced the survival of both of these osteosarcoma cell lines in a concentration-dependent manner. We detected Annexin V and PI double positive Saos-2 cells upon 5 and 35 nm AgNP expositions, consequently these nanoparticles can induce apoptosis in the absence of p53 as well. The extent of caspase activation reflected in procaspase 3 cleavage and confocal microscopic analysis of cell morphology further confirmed the induction of apoptotic mechanisms in both cell lines upon AgNP administrations (Fig. 2). Additionally, decreased proliferation activity was detected in both types of osteosarcoma cells after AgNP treatments, further verifying that AgNPs are able to induce cancer cell growth in a p53 independent way as well (Fig. 1g).

As mitochondrial stress can lead to apoptosis directly, without the involvement of p53, we investigated whether in AgNP-treated osteosarcoma cells mitochondrial functions have been modified. Upon AgNP application, loss of mitochondrial membrane potential, increased leakage of cytochrome c protein into the cytoplasm and elevated levels of ROS were detected both in p53-proficient U2Os and in p53-deficient Saos-2 cells, indicating that AgNPs induce mitochondrial stress in both cell lines (Fig. 5). Since ROS generally induce p53 activation, we examined whether p53 functions were activated in AgNP-treated U2Os cells. Due to AgNP expositions we observed the stabilisation and elevated transactivating capability of p53 (Fig. 4a,c). Additionally, we detected increased *p53*, *p21*, *bax*, *caspase 3* and decreased *survivin* mRNA levels, further confirming the stimulation of p53 signalling upon AgNP treatments (Fig. 4d). Finally, the observed decreased viability of AgNP-treated Saos-2 cells, which express p53 ectopically gave a further support to the idea that p53 contributed to AgNP-induced apoptosis (Fig. 4e).

Although p53 signalling in U2Os cells is stimulated due to AgNP treatments, the obtained viability data on p53-lacking Saos-2 cells suggest that the AgNP-induced apoptosis can be the result of p53-independent events such as the previously detected mitochondrial dysfunction. Since we identified AgNPs of smaller and bigger sizes on the cell surface and in the cytoplasm as well (Fig. 3), but detected no particles within mitochondria and nuclei, it can be hypothesised that silver ions released from the nanoparticles are responsible for the observed mitochondrial stress in both p53-expressing and in p53-deficient osteosarcoma cells. Altogether our results suggest that the primary inducer of the AgNP-provoked cancer cell apoptosis is the mitochondrial stress while the activation of p53 signalling pathway is only a secondary effect.

AgNPs of 5 nm and 35 nm sizes, in the applied concentrations had approximately the same impact on all the examined cellular events including caspase 3 activation, proliferation activity, p53 induction, loss of mitochondrial function and generation of ROS (Figs 1c–g, 2, 4 and 5). Taken together, our results show that the differently sized AgNPs used in this study have common cellular targets and induce identical apoptotic pathways and act primarily through mitochondrial stress.

As a conclusion, by this study we provide further support to the potential application of AgNPs in cancer therapy. We demonstrate here, that both 5 nm and 35 nm sized AgNPs are taken up by osteosarcoma cells and induce cell death in U2Os and in Saos-2 cells via identical pathways. We also show that differently sized AgNPs are able to kill osteosarcoma cells irrespective of the presence or absence of p53. Although AgNP treatments activate p53 in wild type p53-containing osteosarcoma cells, our data indicate that AgNPs can induce mitochondrial stress, which can also drive cancer cell apoptosis in a p53-independent manner.

Since elimination of p53 function is one of the most commonly observed impairments in human cancers, the fact that AgNPs are inducers of p53-independent apoptotic mechanisms, enhances their potential and renders them attractive novel candidates for the rational design of therapeutically useful anticancer agents.

## Methods

### Synthesis and characterization of citrate-coated AgNPs

Citrate-stabilized silver nanoparticles were synthetized by a modified Lee-Meisel hydrothermal reduction method according to Wan et al^53^. The particle size distribution and surface charge (zeta potential) of the obtained silver nanoparticle preparations and of AgNPs in serum free as well as in full culture media were assessed by a Zetasizer Nano Instrument (Marveln, Worchestershire, UK), where AgNP dispersions were dissolved in DMEM medium containing 2 mM L-glutamine, 0.01% streptomycin, 0.005% ampicillin devoid of serum or supplemented with 10% FBS and were analysed 24 h after preparation. The optical absorbance spectra of the citrate-stabilized AgNPs were collected by a UV–VIS spectrometer (Ocean Optics DH-2000-BAL) using 10.0 mm path length quartz cuvettes. UV–VIS absorption spectra of citrate-coated AgNP suspensions were collected over a wavelength range of 250–900 nm.

### Cell culture and viability assay

U2Os and Saos-2 cells were purchased from ATCC and maintained in low glucose DMEM in a 5% CO_2_ atmosphere at 37 °C. Saos-2 medium was supplemented with 5% FBS, while U2Os cells were cultured in medium containing 10% FBS. Both culture media contained 2 mM L-glutamine, 0.01% streptomycin and 0.005% ampicillin.

To measure cell viability upon AgNP or cisplatin (Accord Healthcare) treatments, MTT mitochondrial activity assay was performed. Cells were seeded into 96 well plates (10^4^ cells/well) and treated with AgNPs on the following day. After treatment with AgNPs cells were washed with PBS and incubated with culture medium containing 0.5 mg/ml MTT reagent (Sigma-Aldrich) for two hours at 37 °C. Formazan crystals were solubilized in DMSO and absorbance was measured at 570 nm using a SPECTROstar Nano plate reader. Absorption corresponding to the untreated control samples was considered as 100%. MTT assays were performed using four biological replicates and experiments were repeated at least three times.

### BrdU cell proliferation assay

Cell proliferation was determined according to the level of the incorporated 5-bromo-2′-deoxyuridine (BrdU). 1000 cells/well were seeded into 96 well plates and AgNP treatment was performed on the following day. Cells were exposed to AgNPs for 24 h and BrdU labelling solution was added to the culture medium two hours before the end of the treatment. Cells were fixed and the level of the incorporated BrdU was determined using Cell Proliferation ELISA BrdU assay kit (Roche) following the instruction of the supplier. Proliferation activity of the untreated cells at 0 time point was considered as 100%. BrdU assays were performed using four biological replicates and experiments were repeated at least three times.

### Scanning (SEM) and Transmission Electron Microscopy (TEM)

The morphology of synthetized nano-particles was characterized and their primary size was evaluated by TEM using a FEI Tecnai G2 20 x microscope at an acceleration voltage of 200 kV. Based on TEM images size distribution of AgNPs was calculated by ImageJ software.

For TEM imaging of biological samples 10^5^ cells were seeded onto 0.4 μm pore polyester membrane inserts (Corning) placed in a 6 well plate. Cells were left to grow until the following day when they were treated with AgNPs for 24 hours and carefully washed and fixed in 4% glutaraldehyde in PBS for 2 hours and subsequently embedded in gelatine (2% gelatine in PBS). The obtained specimen was sliced to 1–2 mm cubes, which were further embedded in epoxy (Epon 812, EMS, PA 19440) by a routine TEM sample preparation protocol. Semi-thin sections of 1 μm were prepared to identify the cell monolayer. Blocks were trimmed, thin sections of 70 nm were obtained and stained with uranyl and lead solutions. Images were captured by a Philips CM10 electron microscope using 100 kV voltage. TEM micrographs were taken by a Megaview G2 digital camera (ITEM, Olympus Soft Imaging Solution GmbH, Münster).

For SEM analysis cells were seeded and grown onto plastic coverslips (Sarstedt) placed in 6 well plates and exposed to AgNPs on the following day. After 24 hour treatment, cells were washed and fixed overnight at 4 °C using 2.5% glutaraldehyde (dissolved in modified Sörensen buffer, pH 7.6). The samples were dehydrated by solutions containing increasing percentage of ethanol in water (50%, 70%, 80%, 90%, 95%, 98%, 100%, for 15 min each), followed by a series of tert-butanol:ethanol mixture (1:2, 1:2, 2:1 volume ratio) at room temperature. Then the cells were incubated with tert-butanol overnight at 4 °C and were finally lyophilised. The coverslips were mounted on specimen stubs using electrically conductive double-sided adhesive tape and the sample preparation was completed by a thin (4–5 nm) metal gold-palladium coating needed to decrease charging artefacts and radiation damage of the biological sample. SEM imaging was performed by a Hitachi S4700 electron microscope using 10 kV accelerating voltage and 10 μA emission current. EDS measurements were carried out with a Röntec QX2 EDS detector installed on SEM using 20 kV accelerating voltage and 10 μA emission current.

### Apoptosis detection

For studying the cellular morphology to identify typical apoptotic features, cells were cultured on glass coverslips and treated with AgNPs. Treated cells were washed with PBS and fixed on room temperature with 4% paraformaldehyde. Following permeabilisation with 0.3% Triton-X-100 cells were blocked in 5% BSA solution. For detection of tubulin fibres slides were incubated with anti-tubulin (Sigma-Aldrich) antibody in 1:1000 dilution and with anti-mouse Alexa 647 fluorophore-conjugated secondary antibody. Cells were washed twice in PBST and stained with DAPI solution in 1:1000. Samples were visualised by an Olympus FV10i confocal microscope. The area of cell nuclei and of the cytoplasms were determined using ImageJ software.

For the visualisation of cleaved caspase 3, fixed, permeabilised and blocked cells were incubated with cleaved caspase 3 (Asp175) specific antibody (Cell Signaling) followed by incubation with Alexa 647 fluorophore conjugated secondary antibody. Nuclei were stained with DAPI and samples were examined with an OLYMPUS BX51 microscope equipped with Olympus DP70 camera. The number of cleaved caspase 3 containing cells was determined using ImageJ software.

Activation of apoptosis was also shown on the basis of AnnexinV/PI staining of cells. 3 × 10^5^ cells were seeded into the wells of 6 well plates and treated with AgNPs on the following day for 24 h. Cells were washed with PBS, trypsinised, collected by centrifugation and re-suspended in AnnexinV Binding Buffer. Cells were stained with Alexa 488-conjugated AnnexinV and PI according to the guideline of the manufacturer (Life Technologies). Fluorescent intensities were determined using a FACScalibur flow cytometer by measuring 10.000 cells and FACS data were analysed by FlowJo V10 software. Experiments were repeated three times using at least two biological replicates.

### Western blotting

For western blot analysis cells were lysed in sonication buffer (50 mM TRIS, 2 mM EDTA, 0.5 mM DTT, 50 mM NaCl, 1x PIC) and the obtained extract was used as whole cell lysate. Cell debris including mitochondria were pelleted by 13 000 rpm centrifugation from whole cell lysates and supernatants were used as cytoplasmic fractions. Whole cell lysates and cytoplasmic extracts were boiled in protein loading buffer and samples containing 20 μg protein were resolved on SDS-PAGE and transferred to nitrocellulose membrane (Amersham). Membranes were blocked with 5% non-fat milk-TBST overnight and washed three times with TBST containing 0.01% Tween. p53 specific antibody (DAKO) was used in 1:2000 dilution, cytochrome c specific antibody (Abcam) was applied in 1:500, M2 anti-FLAG antibody (Sigma-Aldrich) and anti-actin antibody (Sigma-Aldrich) was used in 1:2500 and in 1:5000 dilution, respectively. Following three washing steps with TBST membranes were incubated with rabbit anti-mouse or goat anti-rabbit (DAKO) horseradish peroxidase-conjugated secondary antibodies. Membranes were developed using ECL reagent (Millipore) following the guidelines of the manufacturer and chemiluminescent signal was detected by a C-DiGit Blot Scanner (LI-COR).

### Transient transfection and Luciferase Reporter Assay

For transient transfection 3 × 10^5^ cells/well were seeded into 6 well plates. 1 μg of pGL2-mdm2-promoter-Luc reporter plasmid^54^ was transfected into each sample using ExGen (Fermentas) and TurboFect (Thermo Scientific) transfection reagents for U2Os and for Saos-2 cells, respectively, according to the guidelines of the suppliers. 1 μg of pCDNA3-p53-FLAG plasmid expressing a FLAG tagged p53 was transfected into Saos-2 cells cultured in 24 well plates. 24 h after transfections the medium was replaced by AgNP containing culture medium. To perform luciferase assay, AgNP-treated cells were collected by centrifugation and luciferase activity was determined from cell lysates using a Luciferase assay kit (Promega) following the manufacturer’s instructions. Total protein content of the samples were determined using Bio-Rad Protein Assay reagent and bioluminescent signal was detected by an Orion L Microplate Luminometer and corrected according to the protein content of each sample. Transfections were repeated three times using three independent biological replicates.

### RT-qPCR

For RT-qPCR analysis 6 × 10^5^ cells were seeded into 6 cm diameter culture plates and treated with AgNPs on the following day for 24 h. By the end of the AgNP expositions total RNA was isolated using RNeasy Mini Kit (Qiagen) according to the guideline of the manufacturer. Taqman Reverse Transcription Reagent (Applied Biosystems) was used to generate cDNA using 2 μg of RNA and relative levels of *p53*, *p21*, *bax*, *survivin*^55^, *p21* and *caspase 3* transcripts were quantified by a Pico Real-Time qPCR System (Thermo Scientific) using gene specific primers (Table S2) and SYBR Green PCR Master Mix (Applied Biosystems). Ribosomal 18S RNA level was measured to calculate relative amount of each transcript using ΔΔCt analysis. RT-qPCR measurements were carried out at least three times using cDNA obtained from three independent experiments.

### JC-1 staining

To measure mitochondrial membrane potential, cells were seeded into 6 well plates at 3 × 10^5^ cells/well density and treated with AgNPs on the following day. At the end of the treatment AgNP containing media were replaced by 10 μg/mL JC-1 (LifeTechnologies) containing DMEM. After 15 min incubation at 37 °C staining medium was replaced by culture medium and cells were visualised by an OLYMPUS BX51 microscope equipped with Olympus DP70 camera using the same exposition time for all samples. By ImageJ software red to green fluorescent ratio of each sample was determined. Experiments were repeated three times using at least three independent biological replicates.

### ROS staining

DCFDA staining assay was performed to measure the total intracellular ROS level upon AgNP treatments. For this, cells were seeded on glass coverslips in 6 well plates (3 × 10^5^ cells/well) and treated with nanoparticles on the following day. Cells were washed carefully with pre-warmed PBS and incubated with culture medium containing 10 μM DCFDA (Sigma-Aldrich) in dark for 20 min at 37 °C. Cells were washed two times with PBS and fluorescent signals were visualised by an OLYMPUS BX51 microscope equipped with Olympus DP70 camera. For capturing images the same exposition time was applied for all samples. Fluorescent intensity of the samples was analysed using ImageJ software. Measurements were repeated three times using at least three independent biological replicates.

### Statistics

GraphPad Prism 6 Software was used in order to carry out statistical analysis and graphical visualization of the obtained raw data.

## Additional Information

**How to cite this article**: Kovács, D. *et al*. Silver nanoparticles defeat p53-positive and p53-negative osteosarcoma cells by triggering mitochondrial stress and apoptosis. *Sci. Rep.* **6**, 27902; doi: 10.1038/srep27902 (2016).

## Supplementary Material

Supplementary Information

## Figures and Tables

**Table 1:** Calculated IC_50_ values for U2Os and Saos-2 cells after 5 and 35 nm sized AgNP treatments.

Cell line	Treatment (h)	IC50 ± SD (μM)	
		5 nm AgNP	35 nm AgNP
U2Os	24	30.61 ± 2.65	85.47 ± 8.17
	48	15.52 ± 1.49	47.03 ± 4.85
Saos-2	24	20.63 ± 1.087	88.47 ± 6.40
	48	13.94 ± 0.55	45.12 ± 3.24
