# Supplementary material for: Silver nanoparticles defeat p53-positive and p53-negative osteosarcoma cells by triggering mitochondrial stress and apoptosis
Source: Sci Rep. 2016 Jun 13;6:27902. doi: 10.1038/srep27902 (PMC4904210; doi:10.1038/srep27902)
Supplement: Supplementary Information [file srep27902-s1.pdf]

Supplementary information

Silver nanoparticles defeat p53-positive and p53-negative osteosarcoma cells by triggering mitochondrial stress and apoptosis

Dávid Kovács[1], Nóra Igaz[1], Csilla Keskeny[1], Péter Bélteky[2], Tímea Tóth[2], Renáta Gáspár[3], Dániel Madarász[2], Zsolt Rázga[4], Zoltán Kónya[2, 5], Imre M. Boros[1, 6], Mónika Kiricsi[1, *]

[1] *Department of Biochemistry and Molecular Biology, University of Szeged, Középfasor 52, H-6726, Szeged, Hungary*

[2] *Department of Applied and Environmental Chemistry, University of Szeged, Rerrich Béla tér 1.*
H-6720, Szeged, Hungary

[3]*Department of Biochemistry, Faculty of Medicine, University of Szeged, Dóm tér 9. Szeged, H-6720, Hungary*

[4] *Department of Pathology, University of Szeged, Állomás utca. 2. H-6720, Szeged, Hungary*

[5]*MTA-SZTE Reaction Kinetics and Surface Chemistry Research Group, Rerrich Béla tér 1, H-6720, Szeged, Hungary*

[6]*Institute of Biochemistry, Biological Research Center of the Hungarian Academy of Sciences, Temesvári krt. 62, H-6726, Szeged, Hungary*

Corresponding author (*):

Mónika Kiricsi, PhD

Department of Biochemistry and Molecular Biology, University of Szeged

Közép fasor 52, Szeged, Hungary H-6726 E-mail: kiricsim@bio.u-szeged.hu

Supplementary Figure S1

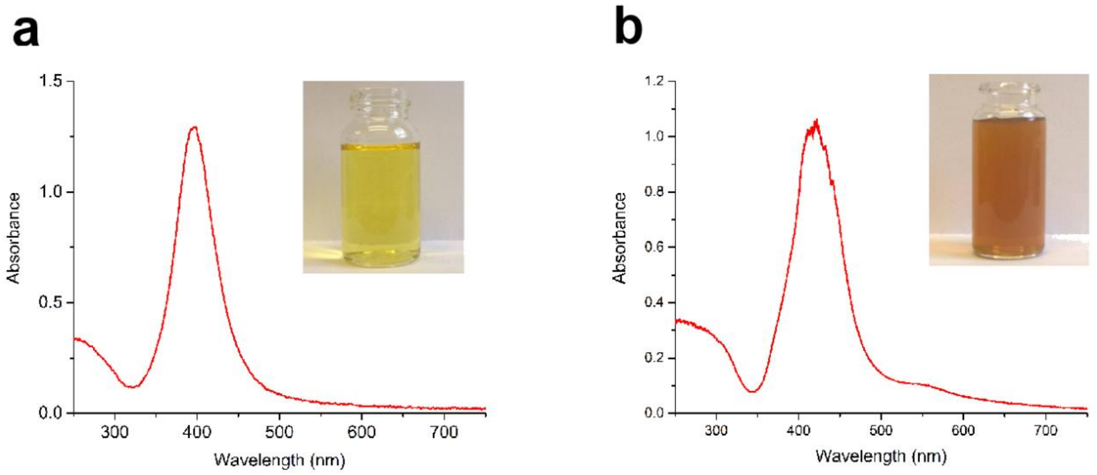

UV-VIS spectra and photos of 5 nm (a) and 35 nm (b) sized silver nanoparticles. The spectra of the AgNP colloidal solutions have a characteristic peak around 400 nm which can be attributed to the surface plasmon resonance. A minor red-shift of the absorption maximum can be observed as the AgNP size increases.

Supplementary Figure S2

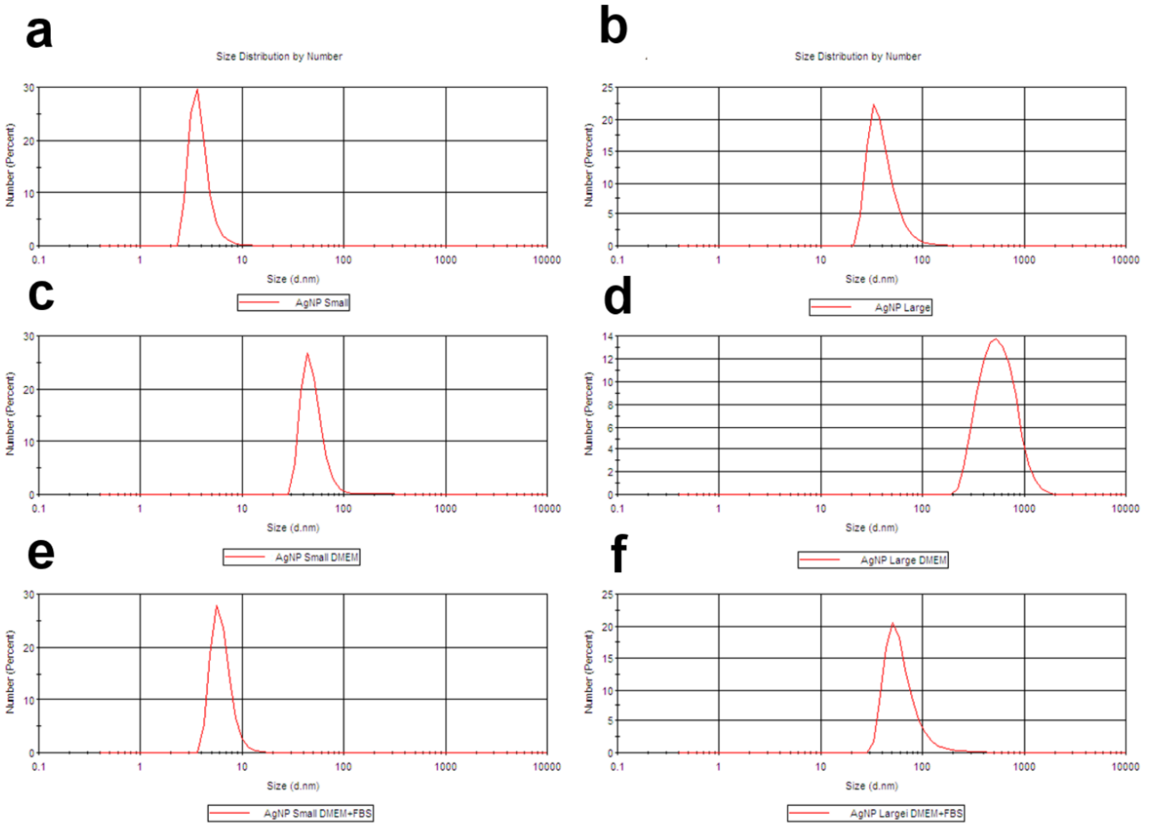

DLS analysis of 5 nm and 35 nm AgNPs in different media. The size distributions of the as-synthesised 5 nm **(a)** and 35 nm **(b)** nanoparticles in aqueous solution were assessed by DLS measurements. Size distributions correlate with the data obtained from TEM image analysis (**Fig. 1b**). In serum free DMEM both smaller **(c)** and larger **(d)** sized particles formed aggregates as significant increase in particle size was detected by DLS. 10% serum containing DMEM inhibited aggregation as both 5 nm **(e)** and 35 nm **(f)** AgNPs manifested DLS spectra similar to those obtained for AgNPs in aqueous solutions. A shift of only a few nanometres was observed due to the protein corona formation on the particle surface.

Supplementary Table S1. Physico-chemical parameters of 5 and 35 nm sized AgNPs.

Parameter	5 nm AgNP	35 nm AgNP
Size ± SD (nm)	4.6 ± 1.52	34.73 ± 8.69
Zeta potential (mV)	-41.93	-43.56
Zeta potential in serum free culture medium	-23.63	-23.10
Zeta potential in serum containing culture medium	-12.58	-14.05

Supplementary Table S2. Primer sequences used for RT-qPCR analysis.

Target	FWD primer	REV primer
18S RNA	5’AAACGGCTACCACATCCAAG 3’	5’ CGCTTCCAAGATCCAACTAC 3’
p53	5’ CCCTTCCCAGAAAACCTACC 3’	5’ CTCCGTCATGTGCTGTGACT 3’
p21	5’ CAGCAGAGGAAGACCATGTG 3’	5’ GGCGTTTGGAGTGGTAGAAA 3’
bax	5' TGCTTCAGGGTTTCATCCAG 3'	5' GGCGGCAATCATCCTCTG 3'
survivin	5' AGAACTGGCCCTTCTTGGAGG 3'	5' CTTTTTATGTTCCTCTATGGGGTC 3'
caspase 3	5’ ACATGGCGTGTCATAAAATACC 3’	5' CACAAAGCGACTGGATGAAC 3'
